# Comparative efficacy of LifeVac® and Heimlich maneuver in simulated airway obstruction

**DOI:** 10.1016/j.jped.2025.02.002

**Published:** 2025-03-21

**Authors:** Maria Lucia S. Hristonof, Marina C. Amantéa, Fernando J. Lazzaretti, Marina M. Bernardes, Luiza F. Xavier, Sérgio Luis Amantéa

**Affiliations:** a Pontifícia Universidade Católica do Rio Grande do Sul (PUCRS), School of Medicine, Porto Alegre, RS, Brazil; b Pontifícia Universidade Católica do Rio Grande do Sul (PUCRS), Infant Center Research Group, Porto Alegre, RS, Brazil

**Keywords:** Asphyxia, Heimlich manoeuvre, Abdominal thrust, Airway obstruction

## Abstract

**Objectives:**

Foreign body airway obstruction is a significant cause of morbidity and mortality, especially in infants and young children. This study aims to compare the efficacy of the Heimlich maneuver and LifeVac® in a simulated environment.

**Methods:**

A prospective experimental study was conducted using the Choking Charlie (Laerdal®) mannequin, which simulates the trunk from an adult male and is considered suitable for simulating choking events in young children. The study involved four operators: one Pediatric Advanced Life Support (PALS) instructor and professor of Trauma and Emergency Medicine, along with three members of the university's Pediatric Academic League, all previously trained in Basic Life Support (BLS). The primary outcome was the success rate of foreign body removal. Intracavitary pressures generated during the maneuvers were measured using a digital manometer.

**Results:**

A total of 200 anti-choking maneuvers were performed, and both techniques successfully relieved airway obstruction in all cases. The LifeVac® device generated significantly lower intracavitary pressure differentials compared to the Heimlich maneuver (*p* < 0.000). Additionally, both techniques exhibited significant variability in applied pressure among different examiners (*p* < 0.000).

**Conclusions:**

Both the Heimlich maneuver and LifeVac® are effective in relieving foreign body airway obstruction when performed by specialists in a simulated environment. Heimlich generated higher positive pressure gradients, while LifeVac® produced lower negative pressure gradients.

## Introduction

Foreign body aspiration into the respiratory tract is associated with significant morbidity and mortality and is a major cause of accidental death worldwide.^1^, ^2^, ^3^, ^4^ Choking injuries are a major contributor to morbidity and mortality among young children and produce a substantial public health burden. Infants and children under three years old account for 75% of the victims in the pediatric population.^5^ The impact on the pediatric population ranges from acute and temporary consequences to chronic manifestations and permanent sequelae.^6^^,^^7^

Foreign Body Airway Obstruction (FBAO) accompanied by asphyxia is considered a medical emergency, and rescue treatment may include abdominal thrusts (Heimlich maneuver), chest compressions, and back blows.^8^^,^^9^ These maneuvers aim to increase subdiaphragmatic pressure, expelling the foreign body from the airway.^9^ However, the evidence supporting these techniques is limited, and their use has been linked to various traumatic complications.^10^^,^^11^

**Figure 1:**
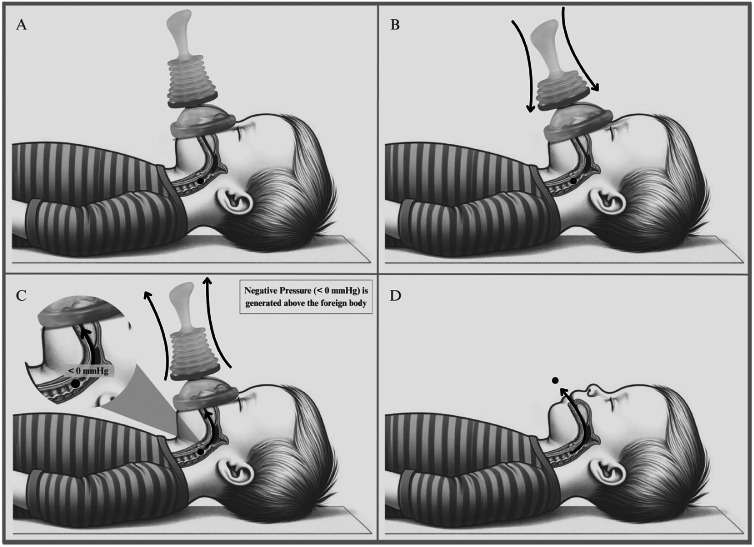
Sequential steps for airway obstruction relief using the LifeVac® device in a pediatric patient. Fig. 1 shows the sequence of using of the LifeVac® device to clear airway obstruction in a child. (A) Positioning the device over the child's mouth and nose, ensuring an airtight seal. (B) Pressing the plunger down to create positive pressure in the airways. (C) Quickly pulling the plunger up to generate negative pressure, dislodging the obstructive object. (D) Successful removal of the object from the airways, restoring the child's breathing ability.

Recently, anti-choking suction devices have emerged as a potential alternative for FBAO treatment. Unlike classical anti-choking maneuvers, which generate positive pressure in the airway, these devices displace the foreign body through negative pressure suction. LifeVac® is one such device that generates negative pressure to assist asphyxiated patients (Figure 1). According to the manufacturer, this device is portable, easy to operate, and does not require an external power source. Despite its biological plausibility, the literature on LifeVac®'s performance is sparse, with most studies involving experimental models or case reports and series.^2^^,^^12^, ^13^, ^14^, ^15^

Experimental studies conducted with the LifeVac® device using mannequins and cadavers have shown promising results. Juliano et al. conducted an experiment on an adult human cadaver using clay to simulate a food bolus obstruction, achieving success in 98% of cases (one attempt) and 100% with an additional attempt.^14^ Lih-Brody et al.^16^^,^^17^ demonstrated LifeVac®'s efficacy at 94% (one attempt), 99% (two attempts), and 100% (three attempts) using different mannequin models.

There are limited real-life clinical studies on the device. Between 2014 and 2020, only 22 cases of LifeVac® use were reported, all of which successfully removed the foreign body within three attempts without side effects.^5^ A prospective observational study of 157 LifeVac® cases from 2021 to 2023 reported nearly universal success but noted 10 adverse events potentially related to the device.^18^ A systematic review indicated insufficient robust evidence to support or discourage the device's use.^9^

This study aims to evaluate the efficacy of disobstruction maneuvers (Heimlich and LifeVac®) in a simulated mannequin scenario and compare the intracavitary pressures generated by these techniques.

## Methods

### Study design

A prospective experimental study was conducted. Four researchers of different genders and age groups performed sequential disobstruction maneuvers for FBAO. The experiment was conducted on a mannequin in the realistic simulation laboratory of the Pontifical Catholic University of Rio Grande do Sul (PUCRS).

### Participants

The group of operators included a Trauma and Emergency professor and three students from the University's Pediatric Academic League, all previously trained in Basic Life Support (BLS). Therefore, all operators were considered qualified and capable of performing the maneuvers. The study was approved by the local ethics committee (CAAE: 80,343,924.1.0000.5336).

### Simulation model

The model used for the FBAO simulation was the Choking Charlie (Laerdal®) mannequin. It weighs 25 lb (11.3 kg) and measures 40.2 in (102.1 cm) in length and 21.2 in (53.8 cm) in width, simulating the trunk of an adult male. It is also considered suitable for simulating choking events in young children (3–4 years), despite having the characteristics of a larger trunk, because airway obstruction (or choking) has similar mechanics, regardless of size. According to the manufacturer, Choking Charlie's airway system is designed to simulate a realistic obstruction in adults and children. Pediatric training in the area of basic life support uses this mannequin for training in the Heimlich Maneuver from the age mentioned (3–4 years).

The model features anatomical landmarks, including the rib cage and navel, to enhance realism in hand positioning training. The mannequin's oral cavity is fixed in an open position and includes a tongue and dental arch. It is primarily made from high-strength plastics and silicone, providing durability and realism. The exterior has a texture that simulates human skin, offering a realistic feel during maneuvers. The internal components are made of plastic with varying degrees of rigidity to ensure that compression or manipulation actions simulate the behavior of a human body more accurately. The mannequin has an airway system structurally designed to mimic human anatomy, albeit in a simplified form for training purposes. The airway is not a rigid tube as found in other types of simulation mannequins. Instead, it is designed to simulate the human respiratory tract realistically, respecting anatomical proportions and allowing trainees to perform compression maneuvers and other first aid interventions with an appropriate tactile response. The head is adjustable, and the neck can be manipulated to alter the position of the airways, making the training more realistic.

The object used for simulating the foreign body (FB) was the accessory provided by the manufacturer (Bolus - Laerdal®). The bolus used as the FB has a spherical shape and, according to the producer, was designed to simulate food in the airway and weighs 0.88 lbs (0.4 kg). It is made of a compressible material and has an approximate diameter of 2 cm. According to the specifications provided by the company (Laerdal®), the object was designed to generate a complete airway obstruction. The Bolus is made from polyurethane foam, a lightweight, flexible material with a soft texture but capable of providing the necessary resistance to simulate airway obstruction.

### Procedure

Each operator performed both disobstruction maneuvers, i.e., using both the LifeVac® device and the Heimlich maneuver. The sequence of maneuvers (LifeVac® and Heimlich; or Heimlich and LifeVac®) was determined by a draw among the operators. Once the order was defined and the technique designated as "first" by the draw was initiated, the operator had to sequentially perform 25 disobstruction maneuvers. After completing this stage, the same operator had to sequentially perform another 25 disobstruction maneuvers with the technique designated as "second" by the draw. For the same operator, a 10-minute interval was allowed between the two stages.

In each new disobstruction maneuver, regardless of the technique used, the time was standardized. The same research team always prepared the mechanical obstruction in the model with the FB, which was manually positioned during each attempt to ensure total upper airway obstruction during the maneuvers. The FB was positioned past the first point of resistance, using the index finger and applying moderate pressure, creating a complete obstruction of the airway.

The estimated time between applying one maneuver and causing a new airway obstruction in the model was approximately 30 seconds. A 30-second pause was taken between each maneuver, totalling a cycle of approximately one minute between sequential maneuvers in each of the two stages.

### Measurement of intracavitary pressure

To evaluate the negative and positive peak pressures generated during the maneuvers, a digital manometer (Homed MVD 300-U®)^19^ was used, which recorded the peak pressure value obtained in real-time. The manometer has a silicone catheter that transmits the pressure from the point of interest to its sensor. Thus, during maneuvers performed with LifeVac®, the catheter's end should be positioned in the oral cavity above the FB to measure the negative peak pressure generated by the device (LifeVac®). During Heimlich maneuvers, on the other hand, the catheter should be positioned in the mannequin's larynx below the FB to measure the positive peak pressure generated by the Heimlich maneuver. The highest pressure value generated during each maneuver was recorded and transcribed to a standardized form.

### Statistical analysis

Continuous variables were described by mean and standard deviation or median and interquartile range. In cases of sample asymmetry, the Kruskal-Wallis test was used. The Mann-Whitney U test was applied to compare distributions between the Heimlich maneuver and the LifeVac® device. To compare means between groups, one-way Analysis of Variance (ANOVA) with Bonferroni post-hoc was applied. Comparisons within groups were assessed by the Wilcoxon test. The significance level adopted was 5% (*p* ≤ 0.05).

## Results

Throughout the experiment, 200 anti-choking maneuvers were performed. Twenty-five sequential maneuvers for both disobstruction techniques by the four different operators. In all maneuvers, airway obstruction by the FB (Bolus®) was successfully relieved. During the Heimlich Maneuver, the peak positive pressure generated was recorded by the equipment's manometry function, generating a pressure gradient from the zero-pressure level. Similarly, in the LifeVac® maneuver, a pressure gradient was generated, but the peak pressure generated was negative, recorded by the equipment's vacuum measurement function. The measured values of peak pressures (both positive and negative), along with their standard deviations, are reported in the Supplementary Material.

**Figure 2:**
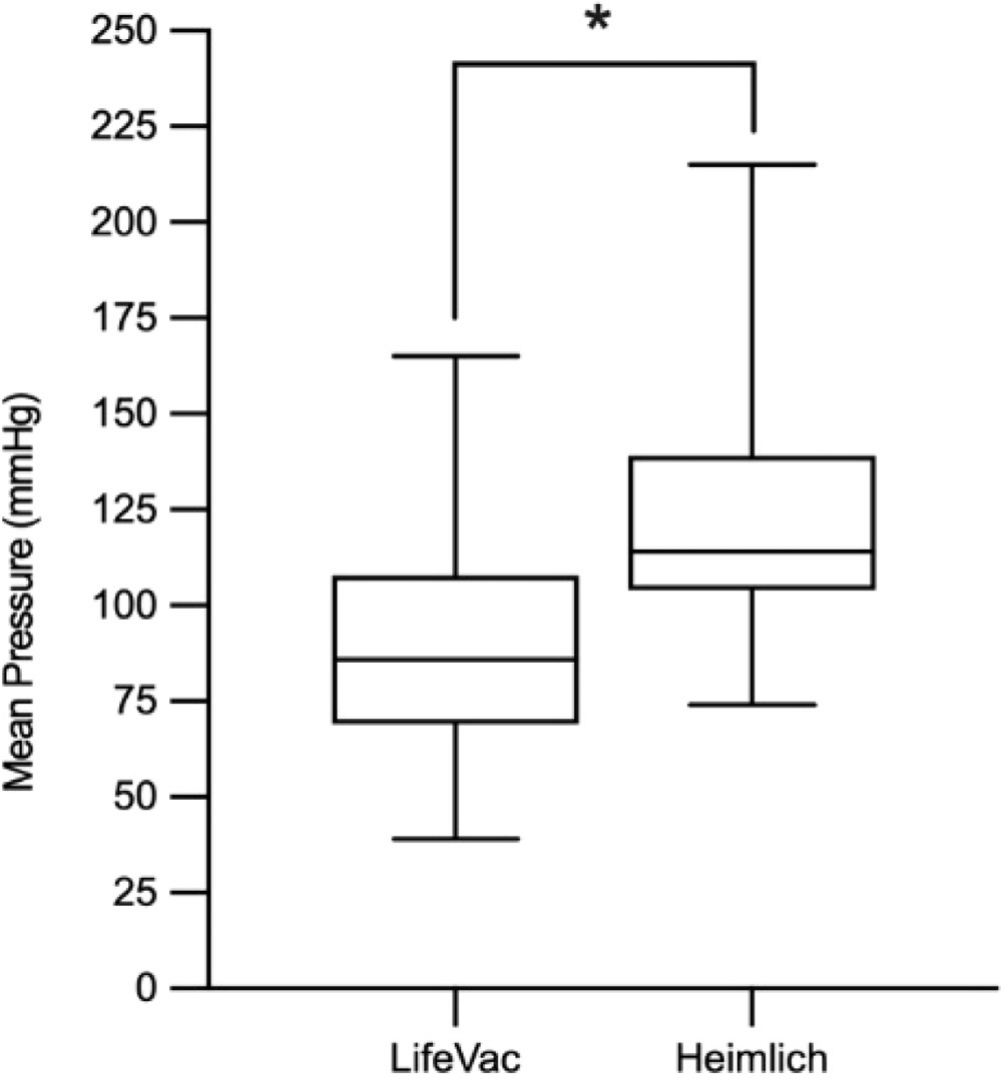
A Single-Baseline Comparison of Negative (LifeVac®) and Positive (Heimlich) Expulsive Airway Pressures. Fig. 2 presents **a** box plot illustrating the mean pressure (mmHg) generated by the LifeVac® device (negative pressure) and the Heimlich maneuver (positive pressure). The data are presented in a single baseline normalization model for standardization, with LifeVac® values not shown below the baseline, as both maneuvers produce a vector in the same direction, facilitating foreign body expulsion. Significant differences (*p* < 0.001, Mann-Whitney U test) between techniques are indicated by a (*).

Significant differences in the pressure gradients (△P) generated by the techniques, compared to the baseline (zero pressure), were observed across all four operators (*p* < 0.001). Specifically, the positive pressure gradients generated by the Heimlich Maneuver, which increase intra-abdominal and intrathoracic pressure, were higher than the negative pressure gradients produced by LifeVac®, which creates suction above the obstruction. The comparison between negative (LifeVac®) and positive (Heimlich) expulsive airway pressures is presented in Figure 2 in a single baseline normalization model to standardize the measurements.

When comparing the pressure gradients obtained by the four operators for both the Heimlich maneuver and LifeVac®, individual pressure differences were observed (*p* < 0.000). The average pressure gradient generated by the examiners applying the two different maneuvers was significantly different.

When comparing the intra-examiner pressure gradient by comparing the values obtained by the Heimlich Maneuver with those obtained using LifeVac®, individual behavior differences were found. Operators 1, 2, and 3 showed differences in the pressure gradients obtained between the two maneuvers (*p* < 0.001), while operator 4 did not show this difference (*p* = 0.478).

## Discussion

In a simulated and controlled environment, with an adult with open mouth model, both the positive pressure-generating maneuver (Heimlich) and the negative pressure-generating maneuver (LifeVac®) were effective in disobstructing the mannequin's airway with a round object, which, according to the producer, was designed to simulate food in the airway. Thus, the previously described biological plausibility for using LifeVac® shows similar performance to the Heimlich maneuver in a controlled experimental environment. This finding is consistent with some reports and small case series, as well as a prospective observational study, that describe success in treating FBAO using the device.^18^

This comparative finding between techniques is significant because, to our knowledge, no existing study, even in a simulated environment, has directly evaluated both techniques while incorporating the measurement of intracavitary pressures.^18^ Our work aligns with the suggestion by Dunne CL and colleagues in a prospective case series, which proposed that pre-clinical studies in a simulated environment comparing disobstruction techniques are valuable. They argued that querying databases would not provide conclusive evidence for the use of these devices due to the rarity of such events and the challenges in their characterization.^18^

In the series by Dunne CL and colleagues, conducted over two years (July 2021–June 2023), the use of two different negative pressure-generating devices was evaluated in 186 adult patients: LifeVac® (*n* = 157/84.4%) and Dechoker® (*n* = 29/15.6%). LifeVac® was the last intervention before airway obstruction relief in 151 of 157 cases.^18^ The performance of the devices was similar, and operators agreed on the ease of applying the technique and the safety of these devices.^18^

In another study, McKinley MJ and colleagues described the use of the LifeVac device in adult patients from 2014 to 2020.^12^ In their series, 39 patients had conditions that put them at risk for dysphagia. In 38 patients, the device resolved the choking incident, and the patients survived.^12^ Although the device successfully removed the obstruction in the 39th patient, as confirmed by paramedics, the patient could not be resuscitated despite CPR maneuvers.^12^

Although the literature reports the ease of application of the device, our observations revealed differences in pressure gradients between techniques and among operators. While the device is generally easy to handle, its efficacy may vary among operators. Despite all maneuvers being effective in dislodging the obstruction, the pressure values generated varied significantly.

Cardalda-Serantes B. and colleagues outlined a study with 43 health science students to resolve FBAO in three simulated mannequin scenarios: 1) using LifeVac®, 2) using Dechoker®, and 3) following BLS protocol recommendations.^20^ The technical compliance rates in the three scenarios and the time needed to complete each maneuver were evaluated.^20^ All scenarios were adequately resolved with the employed techniques.^20^ However, the time difference favoring the use of LifeVac® compared to other maneuvers stands out.^20^ The adequacy rates for the technique at all stages were not different between devices.^20^ They showed adequacy rates of 60% and 80%, respectively, considering the use of LifeVac® and Dechoker®.^20^ This scenario indicates a percentage of operators who do not fully comply with all technical steps when using the devices.

Therefore, we can infer that variations in the final outcomes may arise due to differences in the execution of the techniques by different operators, even when considering the same intervention stage (Heimlich or LifeVac®). The reported ease of using the device may not translate into evaluative outcomes of technique and generated pressure gradient. We believe that regular and systematic training should contribute to better praxis and a consequent approximation of these technique-related outcomes.

As for the observed pressure gradient differences considering the use of LifeVac® compared to the Heimlich Maneuver, we were not surprised. The Heimlich Maneuver is known to be associated with a higher incidence of complications and traumatic events, including vascular, gastroesophageal, and thoracic injuries.^11^, ^12^, ^13^, ^14^, ^15^, ^16^, ^17^, ^18^^,^^21^ Severe complications such as pneumomediastinum, aortic valve rupture, diaphragmatic herniation, aortic dissection, gastric rupture, and splenic rupture have been reported.^11^^,^^21^ Therefore, it is reasonable to expect that a technique generating higher pressure gradients would be associated with a higher occurrence of these complications, even though there may be other causes for these conditions, such as direct trauma during the maneuver.

Our study has some limitations. The primary limitation is the simulated and controlled nature of the experiment, which was conducted on mannequins. Although designed to replicate the human airway, the mannequin's system is simplified for training purposes. This does not fully replicate real-life conditions where the behavior of pressure gradients and the effectiveness of disobstruction techniques might vary. Additionally, the study involved only four operators, all of whom were highly trained and familiar with BLS, representing a small and specialized sample that may not be representative of the general population.

Despite the limitations, our study suggests that airway disobstruction in experimental models using negative pressure-generating anti-choking devices seems promising. However, for real-life scenarios, the results should still be interpreted with caution. Our findings indicate that the pressure gradients generated are lower than those from the Heimlich Maneuver and that there are variabilities in the intracavitary pressures generated by the technique among examiners. Additionally, although it is referred to as easy to apply in most related articles, our findings may suggest the need for greater training.

Both the Heimlich maneuver and LifeVac® are effective in relieving foreign body airway obstruction when performed by specialists in a simulated environment. The Heimlich maneuver generated higher positive pressure gradients, increasing intra-abdominal and intrathoracic pressure, while LifeVac® produced lower negative pressure gradients through suction above the obstruction. Moreover, our findings indicate that the device application may involve certain complexities.

## Conflicts of interest

The authors declare no conflicts of interest.

## Appendix Supplementary materials

Image, application 1
