# Supplementary material for: Comparative efficacy of LifeVac® and Heimlich maneuver in simulated airway obstruction
Source: J Pediatr (Rio J). 2025 Mar 21;101(3):473–8. doi: 10.1016/j.jped.2025.02.002 (PMC12039517; doi:10.1016/j.jped.2025.02.002)
Supplement: Supplementary file 1 [file mmc1.docx]

**JPED-D-24-00607_Supplementary Material**

**Table** **1** Operator-Generated Pressures for Heimlich Maneuver and LifeVac: Individual Attempts, Mean, and Standard Deviation.


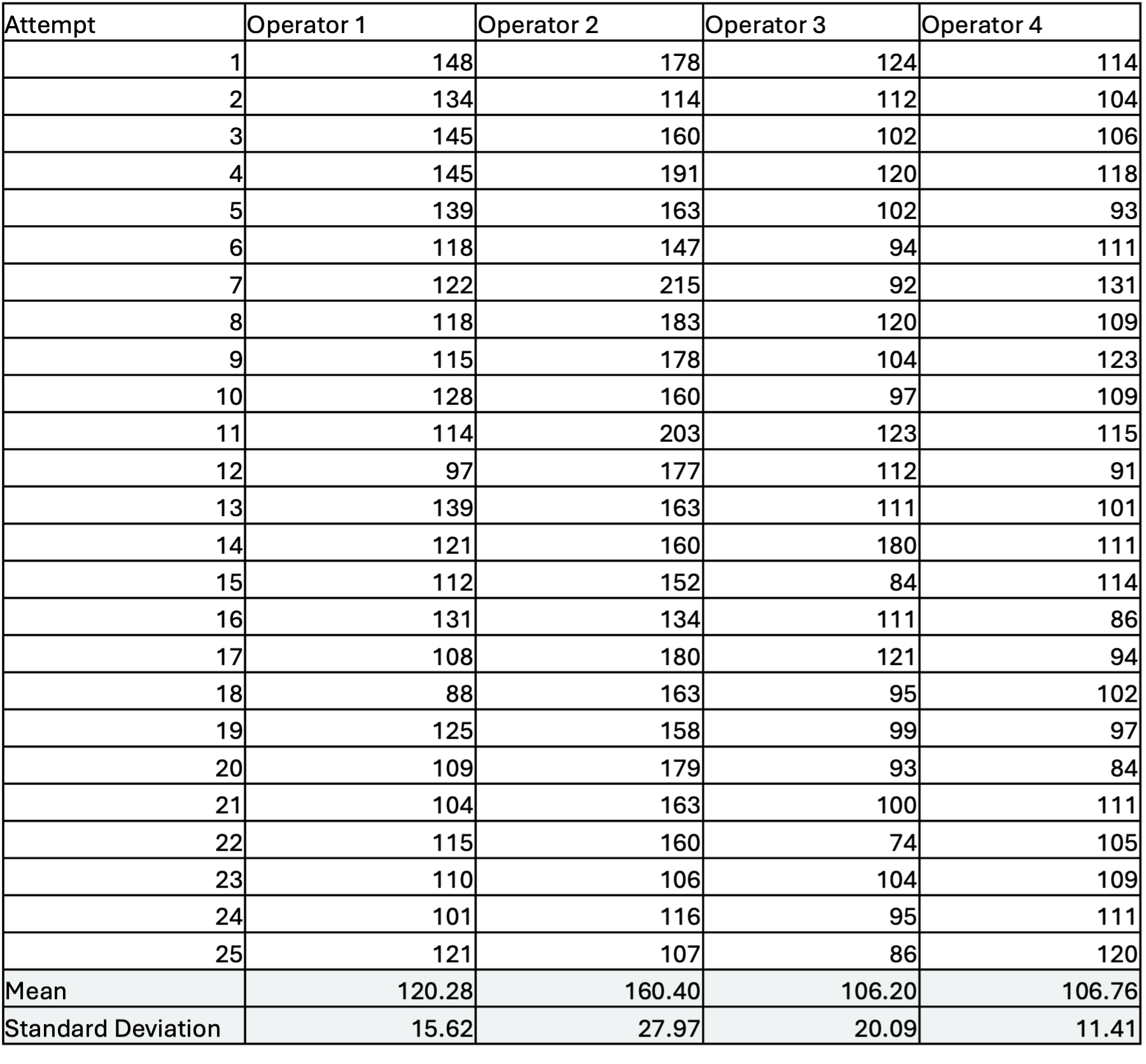

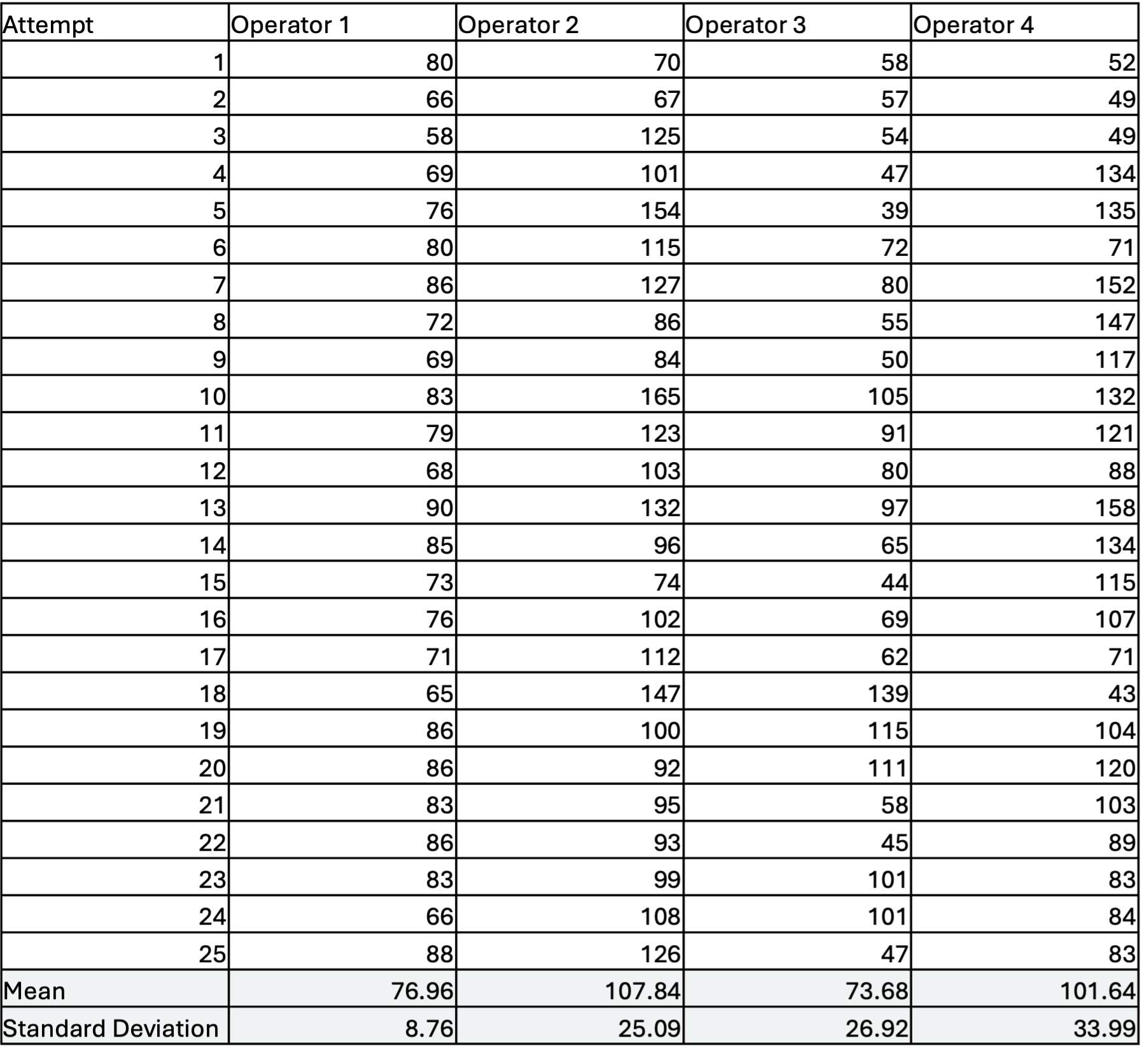
**Heimlich** **ManeuverAttempts** **LifeVac** **Attempts**
